# Using mobile phone data to examine weather impacts on recreational ecosystem services in an urban protected area

**DOI:** 10.1038/s41598-021-85185-7

**Published:** 2021-03-10

**Authors:** Wanggi Jaung, L. Roman Carrasco

**Affiliations:** 1 Division of Social Sciences, Duke Kunshan University, Kunshan, China; 2 Environmental Research Center, Duke Kunshan University, Kunshan, China; 3 Department of Biological Sciences, National University of Singapore, Singapore, Singapore; 4 Campus for Research Excellence and Technological Enterprise, Singapore, Singapore

**Keywords:** Environmental sciences, Environmental social sciences

## Abstract

Mobile phone big data can offer new opportunities for identifying weather impacts on recreational ecosystem services in protected areas. This could be useful to assess how climate change could affect recreational ecosystem services. To explore these opportunities, we utilize mobile phone data and examine impacts of tropical weather (temperature, rainfall, and wind) and holidays on visitor numbers and stay time in an urban protected area in Singapore. These impacts were analyzed by visitors’ home regions and ethnic groups as well. The study results showed that rising temperatures below 31.7 °C had positive impacts on visitor numbers, in contrast to the common perception that cooler temperatures would be always preferred for outdoor activities in a tropical region. Meanwhile, these rising temperatures reduced visitor stay time in the protected area. Rain and wind had limited impacts on visitors. Compared to the weather variables, holidays had bigger impacts on visitors, particularly the Chinese group and those visitors living not close to the protected area. The study results highlight several advantages of mobile phone data application to analyzing weather impacts on public use of urban protected areas.

## Introduction

Monitoring visitors is vital for managing protected areas. As one of the last remaining habitats for wildlife, protected areas not only play an essential role in conserving biodiversity, including those protected areas within human-dominated regions^1^, but also support public well-being by providing recreational opportunities^2^. Recreational activities in nature help visitors improve physical and mental health, and allow visitors to have cultural and spiritual experiences in nature^3–6^. Managing these multiple benefits from protected areas, however, has been challenged by increased visitation globally^7^. Although a large number of visitors represent high recreational benefits from protected areas, high visitation could also result in negative biophysical impacts on protected areas resulting in biodiversity loss^6,8^. Urban protected areas are particularly subject to such tradeoff as their easy accessibility results in massive visitation^9,10^. To holistically manage this tradeoff, therefore, it is critical to monitor visitors to protected areas and understand the factors affecting the visitation^6,7^. One of these key factors is climate change whereby changes in temperatures could either enhance or curb outdoor activities of the public, including visitation to protected areas^11–13^.

Climate change could alter the public’s use of protected areas because outdoor thermal comfort influences their outdoor activities^7,14,15^. Analyzing outdoor thermal comfort is however complex. It is influenced by both physiological and psychological factors^16^. In addition, thermal comfort could differ between indoor and outdoor conditions^17,18^, between dry and wet weather conditions^19^, before and during outdoor activities^20^ and in different outdoor spaces, such as urban parks and public squares^21^. Thermal comfort might even differ among different ethnic groups^15^ as many studies report that different ethnic groups have different preferences and motivations for outdoor recreation^22–26^. The complexity of thermal comfort emphasizes the importance of understanding diverse microclimatic and social factors in investigating the potential impacts of climate change on public use of protected areas.

Mobile phone data have the potential to support examining microclimate impacts on public use of urban protected areas as big data can robustly record the revealed behaviors of large populations over time^27–29^. For example, Jiang, et al.^30^ illustrate the capacity of mobile phone data to identify daily mobility patterns of mobile phone users in comparison with surveyed census data in Singapore. This characteristic of mobile phone data allows analyzing these data to be more cost-efficient than conducting traditional on-site surveys^30^ because surveying a massive population often entails high cost and complex survey administration (e.g. monitoring diverse entrances to protected areas). Consequently, there have been increased big data applications to monitor the visitation to protected areas^7^. Flickr and Instagram have been the dominant sources of these big data^7,31–38^ while some studies rely on GPS tracking devices, smartphone-based GPS devices, and infrared mechanical counters^7,39,40^. Moreover, there are studies applying mobile phone data to examine visitation to other nature-based recreation sites, such as urban parks^41–43^.

To explore the potential of big data to support complex analyses of visitation to protected areas, we applied mobile phone data and analyzed impacts of tropical weather on public use of Bukit Timah region in Singapore, which is an urban protected area. Mobile phone data revealed the number of visitors, their stay time in the area, visitor home regions, and visitor ethnic groups. The main research questions of this study were: (1) *does tropical weather affect public visitation numbers in the studied urban protected area?*; (2) *does tropical weather affect the public’s stay time in the area?; *and (3)* what are challenges and opportunities for applying mobile phone data to analyzing weather impacts on public use of the protected area?* We analyzed impacts of weather and holidays on both visitor numbers and their stay time in the protected area. Visitor numbers were analyzed as weather impacts on visitor decisions to go to the protected area before they experience physical outdoor activities. Visitor stay time was examined as weather impacts on visitor decisions to leave the protected area after having outdoor activities in the area. As weather variables, we analyzed daily maximum temperature, daily total rainfall, and daily mean wind speed. All these variables are strongly connected with outdoor thermal comfort^15,19,44^ and the Tourism Climatic Index^45,46^. We also examined weather impacts on visitors from different home regions and visitors in different ethnic groups. These analyses allowed testing weather impacts on visitors with different sociodemographic factors, such as different distances to the protected area^47–49^ and different cultural backgrounds^15^. Representing all national holidays and weekends, a holiday variable was analyzed in this study because institutional seasonality can have strong impacts on travel behaviors^50^ to be compared with the weather factors. Overall, this study contributes to identifying the potential of mobile phone data to support analyzing complex weather impacts on public use of urban protected areas with regard to various sociodemographic factors.

## Results

**Figure 1:**
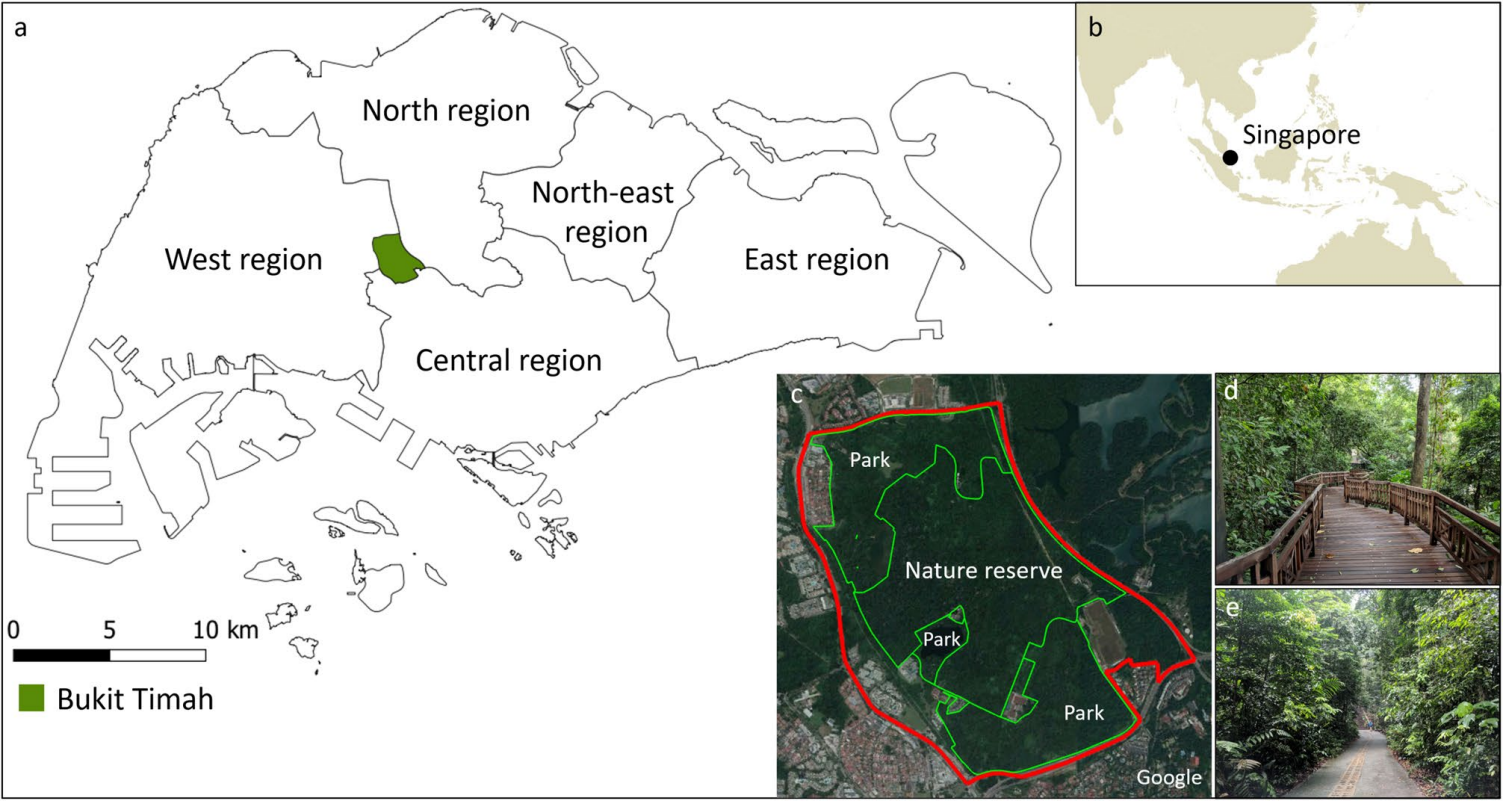
Bukit Timah in Singapore. The area consists of a nature reserve and parks. The regions were defined based on the Master Plan 2014 Region Boundary from the Urban Redevelopment Authority in Singapore. (Data sources: (**a**) https://data.gov.sg using QGIS 3.16.0^52^, (**b**) R version 3.4.1^53^ with Package maps^54^, (**c**) ©2019 Google and https://data.gov.sg using QGIS 3.16.0^52^, (**d**) the authors, and (**e**) the authors).

**Figure 2:**
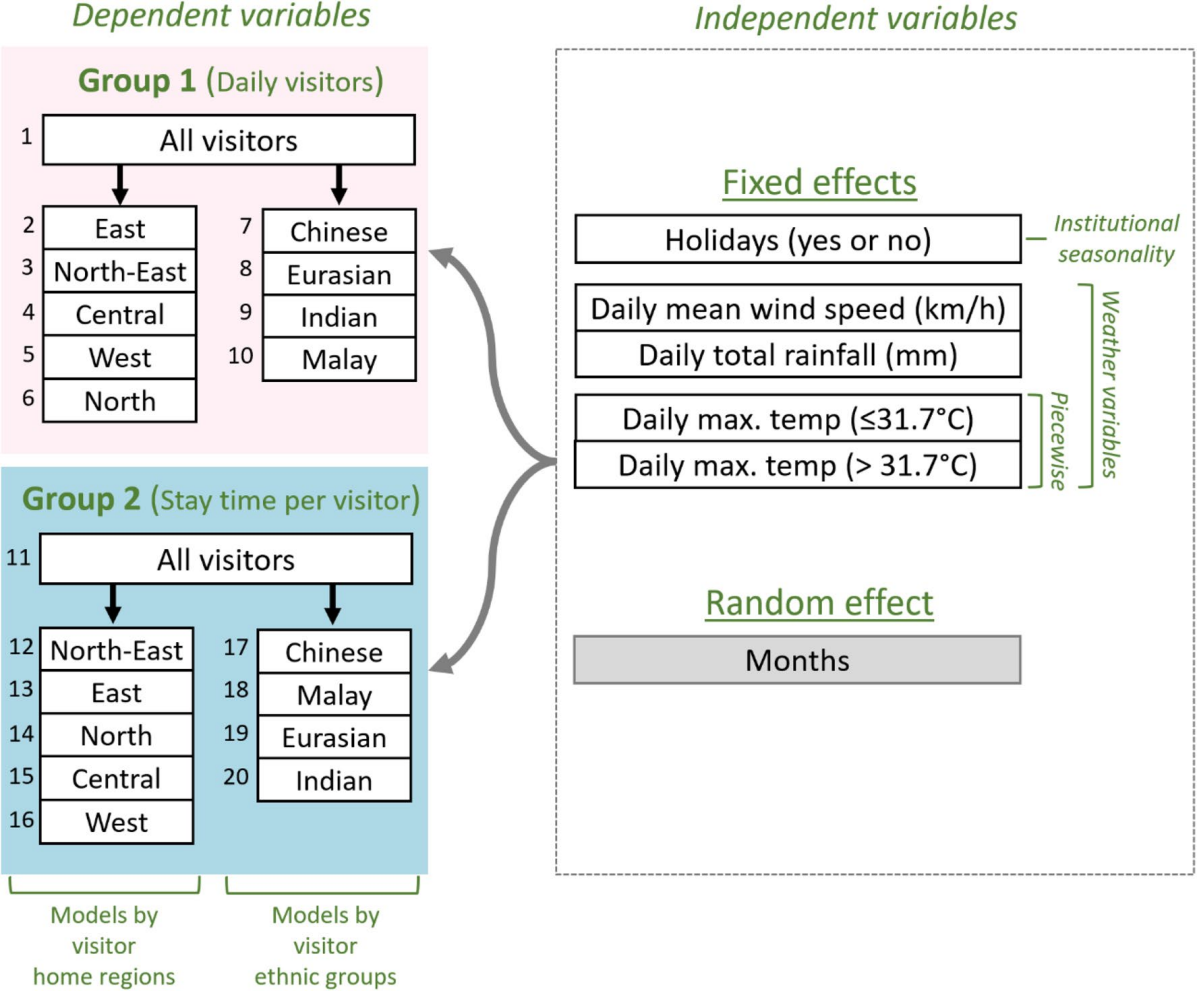
The structure of the generalized linear mixed-effects models in the study. A total of 20 models were established using 20 dependent variables. Group 1 models were used to analyze visitor numbers, indicating weather impacts on visitors’ decisions to visit the protected area before they experience physical activities in the protected area. Group 2 models were utilized to examine visitor stay time in the protected area, representing weather impacts on visitors’ decisions to leave the protected area after they experienced physical activities in the protected area. (Image source: the authors).

Using mobile phone data, we examined impacts of weather and holidays on the number of daily visitors and their stay time in Bukit Timah in Singapore (Fig. 1). Singapore is an island city-state in a tropical climate^51^, and Bukit Timah includes an urban protected area which is under “Category IV: Habitat/Species Management Area” from the International Union for Conservation of Nature (IUCN)^10^. The mobile phone data were recorded from May to December 2017, capturing more than 850,000 visits to the protected area. These data existed as anonymized count data so that although we could not identify individual visitors, we were able to identify visitors’ home regions (e.g. the numbers of visitors from East, Central, North, North-East, and West regions) and their ethnic groups (e.g. the numbers of Chinese, Eurasian, Indian, and Malay Singaporeans). The weather variables included daily maximum temperature (°C), daily total rainfall (mm), daily mean wind speed (km/h). The holiday variable represented public holidays and weekends (1: yes, and 0: no). We applied generalized linear mixed-effects models to examine impacts of these variables on public use of the protected area. Using a piecewise regression approach, daily maximum temperature was examined as temperatures below 31.7 °C (≤ 31.7 °C) and temperatures above 31.7 °C (> 31.7 °C). This breakpoint (or threshold) was identified by analyzing the Akaike information criterion. Based on visitors’ home regions and ethnic groups, we established multiple models and categorized these models into two groups (Fig. 2). Group 1 included models analyzing weather and holiday impacts on the number of daily visitors. Group 2 consisted of models examining these impacts on visitor stay time in the protected area.

**Figure 3:**
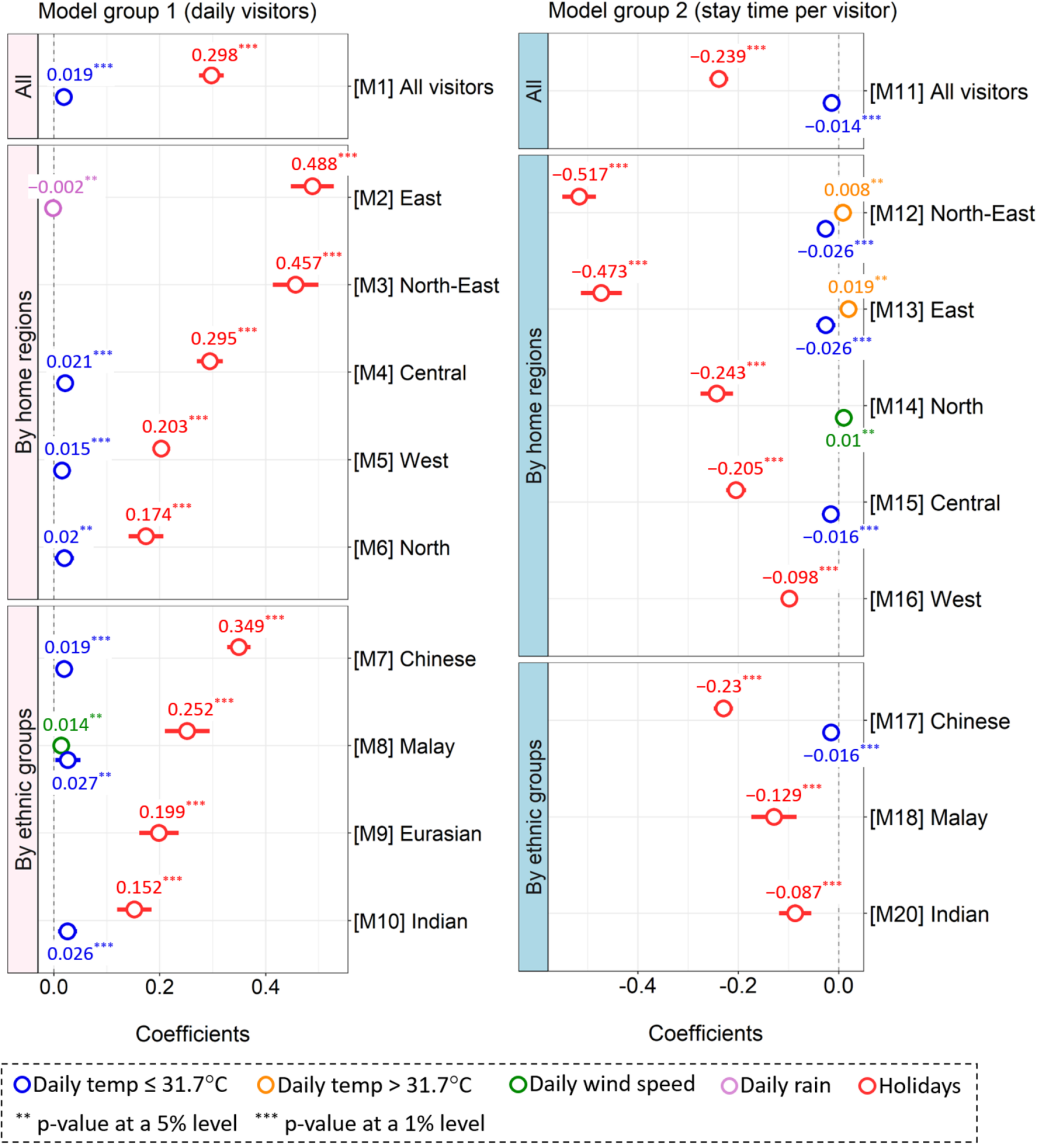
Results of the generalized linear mixed-effects models. Group 1 (M1 to M10) analyzes daily total visitors to the urban protected area. Group 2 (M11 to M20) analyzes stay time per visitor (minutes) in the urban protected area.

Group 1 models showed that the number of daily visitors of the urban protected area was positively affected by the temperature variable with a threshold of ≤ 31.7 °C (Fig. 3). This temperature variable increased visitor numbers (M1) (coefficient: 0.019). However, the degrees of these temperature impacts appeared differently in some regions and ethnic groups. These temperature impacts were not observed from visitors from East Region (M2) and North-East region (M3), as well as the Eurasian group (M9). The coefficients of the temperature variable were not substantially different among these models, regardless of home regions and ethnic groups. Unlike rising temperatures lower than 31.7 °C, the rising temperatures above 31.7 °C had no statistically significant impacts on all models in Group 1. Unlike the temperature variable with a threshold lower than 31.7 °C, daily total rainfall and daily mean wind speed only had limited impacts on visitors from specific regions or ethnic groups. The rainfall variable had slightly negative impacts (− 0.002) on the visitors from the East Region (M2). The wind variable had positive impacts (0.014) only on the Malay group (M8). Compared to all weather variables, the holidays variable had larger positive impacts on visitor numbers (M1 to M10). The coefficients of the holiday variable ranged from 0.152 to 0.488, which were larger than the coefficients of the temperatures below 31.7 °C (from 0.015 to 0.027). These holiday impacts were also observed with visitors from all home regions and ethnic groups. Among the home regions, the largest holiday impacts (0.488) were observed from visitors from the East Region (M2). The smallest impacts (0.152) existed with visitors from the North Region (M6). Among the ethnic groups, the holiday impacts were largest with the Chinese group (M7), while smallest with the Indian group (M10).

Group 2 models demonstrated that stay time per visitor was negatively affected by the temperature variable with a threshold of ≤ 31.7 °C (M11) (Fig. 3). The variable also had negative impacts on the models of the North-East Region (M12: − 0.026), East Region (M13: − 0.026), Central Region (M15: − 0.016), and the Chinese ethnic group (M17: − 0.016). Compared to the temperature variable, however, the rest of the weather variables had limited impacts on the visitor stay time. The temperature variable with a threshold of > 31.7 °C had positive impacts only on the stay time of visitors from the North-East (M12: 0.008) and East Regions (M13: 0.019). The wind variable only had a positive impact on the stay time of visitors from the North Region (M14). Unlike Group 1 models, the rainfall variable did not have any statistically significant impacts on Group 2. In addition, the holiday variable had stronger impacts on all Group 2 models compared to the weather variables. The coefficients of the holiday variable ranged from − 0.517 to − 0.098. These holiday impacts were the largest with visitors from the North-East Region (M12: − 0.517) and the Chinese ethnic group (M17: − 0.23). However, these impacts were the lowest with visitors from the West Region (M16: − 0.098) and the Indian group (M20: − 0.087). All variables of the Eurasian model (M19) were not statistically significant at a 5% level.

## Discussion

### Weather impacts

The mobile phone data analysis revealed multiple weather impacts on public use of the urban protected area in Singapore. Rising temperatures below 31.7 °C had positive impacts on almost all Group 1 models, including the model of all visitors (M1) (Fig. 3). At the average visitation level of 3502 people, the increase in one degree in the all visitors model leads to an expected increase in 67 daily visitors (1.9% increase). Although this result is statistically significant, its public health significance is relatively small in terms of percentage. Considering however the large disease burden associated with mental health (annual prevalence of 6.4% in Singapore^55^, and the potential of nature experiences to reduce this burden, enhancing the nature dose through one extra visitation to nature in 1.9% of the population could however be of interest. For instance, it has been shown that 30 min of nature dose per week can reduce depression by 7%^56^. As such, the effect size could potentially translate in a contribution to reducing depression in 1.3 individuals for every 1000 people. The effect of holidays is, by contrast, of much clear public health significance, with expected increases of 36% in visitation numbers during holidays. The effect of temperature on duration of the visit is also estimated to have a small effect size (0.014) which translates, after back-transformation, into 1.4% less duration of stay in the nature reserve. With an average stay of 240 min, the effect of temperature is only 3.4 min less per visit. Although this is a small reduction in nature dose, it may be significant from a public health perspective. Assuming three visits to green spaces per week, about 10 min of nature dose would be lost per person for one degree of temperature increase. This would represent 8% of the minimum dose of 120 min recommended for good health and wellbeing^57^, 17% of the one-hour weekly dose associated with higher life satisfaction in people connected to nature^58^ and 33% of the 30-min nature dose necessary to reduce depression prevalence by 7%^56^. As such, although the effect sizes of temperature are remarkably small, their potential public health significance may merit further consideration.

The small role of weather on nature reserve visitation may reflect the idiosyncratic situation of Singapore with extremely low variability in terms of temperature and humidity that are expected to be always within a narrow range. This would mean that potential park visitors are unlikely to postpone a visit due to the weather and to factor weather in the planning of park visitation long term. However, the non-negative impact of rising temperatures below 31.7 °C was surprising as this finding was contrary to the common perception that the public in Singapore suffers from hot tropical temperature so that they always prefer cool temperatures for their outdoor activities in Singapore. This finding is inconsistent with the findings from non-tropical countries that park visitors’ thermal tolerance is below 31.7 °C^11,14,40^. However, the result is supported by other thermal comfort studies in Singapore. Yang, et al.^18^ show that the public accepted 26.3–31.7 °C as outdoor operative temperatures in Singapore. Meng, et al. 19 show that cyclers in Singapore considered 29.5–31.5 °C as good outdoor temperatures for cycling. Psychological expectations are also known to affect outdoor thermal comfort^59^, implying that the public in Singapore might expect hot outdoor temperature so that their thermal tolerance could be higher than those in non-tropical countries.

On the other hand, rising temperatures above 31.7 °C did not affect visitation to the protected area (Fig. 3). These results supported that the selected breakpoint (31.7 °C) in the piecewise approach successfully distinguished temperatures with threshold impacts (≤ 31.7 °C). These statistically insignificant impacts of temperatures above 31.7 °C potentially indicate that in Singapore public’s tolerance to high temperatures might be stronger than their tolerance to cool temperatures. However, these study results still cannot confirm that the public would tolerate all temperatures above 31.7 °C. It is expected that temperatures in Singapore would increase by climate change^60^, and it is certain that the public will not tolerate high temperatures at a certain level. Climate change would also affect the threshold temperature impacts beyond those identified in this study as the public’s tolerance to temperatures could change with climate change as well. These possibilities imply a need for future studies on how climate change would influence visitation to protected areas.

Unlike the impacts on visitor numbers, however, rising temperatures below 31.7 °C reduced visitor stay time in the urban protected area (Group 2 in Fig. 3). A potential cause of these impacts might be a reduction of visitors’ thermal tolerance as they experience physical activities in the protected area^17,20^. The result is supported by studies showing that visitors stayed in an urban park longer when their thermal comfort was met^21^ and weather can affect the quality of visitor experiences in a marine park^61^. Moreover, these temperature impacts were particularly observed from the Chinese ethnic group, indicating that different ethnicities might respond differently to the same temperature. An unexpected finding is that rising temperatures above 31.7 °C had positive impacts on stay time of those visitors from the North-East and East Regions. A potential reason might be that, compared to the other regions, these two regions have the longest trip distances to the protected area. In turn, when daily maximum temperature was too high particularly at noon, they might have decided to avoid their home trips during these times and wait in shelters until sunlight decreases. However, further investigation would be required to identify the actual causes of this visitor behavior.

### Holiday impacts

The variable of holidays allowed comparing the weather impacts with other social factors affecting the visitation to the protected area. As institutional seasonality^50^, the holiday variable represented all national holidays and weekends during the study period. These strong holiday impacts observed were consistent with several studies on park visitors^40,62^. These results emphasize that weather impacts represent only some of the factors affecting the visitation to the protected area. In addition to weather impacts, therefore, other social factors should be considered together in managing visitation to protected areas. In this study, the holiday variable could capture some characteristics of visitor home regions and ethnic groups as well. For instance, visitors with longer traveling distances (the East and North-East Regions) usually visited the protected area during holidays (Fig. 3). These impacts of travel distances were supported by the travel cost method in the literature, asserting that travel distances affect the public use of recreational sites^43,47^. In addition, the holiday variable had different impacts on Central, North, and West Regions even though all these regions are adjacent to the projected area. These impacts might reflect different spatial conditions in the regions (e.g. concentration of businesses in the Central Region, and availability of local parks in these regions). The Chinese ethnic group also visited the protected area more frequently during holidays, while the other ethnic groups, including Eurasians, were relatively less affected by holidays. These results were consistent with a study in Chicago showing that Caucasian visitors regularly visited an urban park on weekdays, while Asian visitors used the park more frequently on weekends^26^. During holidays, moreover, visitors’ stay time in the protected area reduced significantly. This might be caused by crowdedness during holidays, which could have negative impacts on visitor preferences^63^. In this manner, the holiday variable demonstrates multiple and complex social factors affecting public visitation to the protected areas, in addition to the weather variables.

### Lessons from mobile phone data application

The application of mobile phone data had limitations, reflecting the limitations of this study as well. First, these data only identified visitors who brought a mobile phone to the protected area and those who are customers of the mobile service provider. Second, the mobile phone data could not indicate direct perceptions of people, which is a common limitation for other types of big data^29^ as well as visitors’ coping strategies related to thermal comfort such as changing clothes^13^. Collection of such data would need to rely on other types of data such as perception surveys and geotagged photographs visually presenting visitor behaviors. Third, it is not possible to apply mobile phone data to analyze thermal comfort indices based on specific personal data, such as public perceptions on weather, or visitors’ skin temperatures^13^. Fourth, these mobile phone data only identified visitors within defined geographical boundaries, such as the zone of Bukit Timah. Owing to this limitation, we could not apply these data to analysis of other nature reserves in Singapore. Fifth, the mobile phone data in this study were not panel data so that the data did not support analyzing impacts of multiple sociodemographic characteristics (e.g. visitor home regions and ethnic groups) as independent variables in the models. Last but not least, mobile phone data were inaccessible to the public, like other big data^29^, even though social media data in certain platforms are openly available for public use. For the same reason, we could obtain mobile phone data for only a particular period of time.

On the other hand, mobile phone data application provided several advantages in analyzing weather impacts on public use of an urban protected area. First, the data represented a massive population of visitors at a national level. The data were capable of reporting behaviors of about 49% of the mobile phone users in 2017 in Singapore^64^ and counted more than 850,000 visits for 8 months. Unlike geotagged photographs, moreover, mobile phone data were not limited to particular visitors who take photos and update them to a particular social media, such as Flickr. Second, the use of mobile phone data was less expensive than the expected costs of surveying such large number of visitors over long time periods^30^. Third, these data were capable of monitoring visitors from multiple entrances of the protected area. A field validation study in Singapore^30^ indicated that these data could identify diverse travelling behaviors of mobile phone users. This capacity enhances the accuracy of visitor counts in the protected area since Bukit Timah presents multiple entrances^10^. Fourth, the data were based on revealed behaviors of visitors over time, instead of relying on visitor memories on past experiences. Unlike surveys asking past experiences of visitors (e.g. population surveys), therefore, mobile phone data are not subject to measurement biases from participant memories. All these advantages highlight the potential and strengths of mobile phone data to support diverse analyses of urban protected areas, including monitoring impacts of weather changes on public use of protected areas.

### Study limitations

There are several limitations of the study. First, Singapore represents one of the urban cities in the tropical regions so that there is a need for future studies on other tropical regions to verify whether park visitors in tropical cities have a high tolerance to high temperature compared to park visitors in non-tropical regions. Second, the mobile phone data might include some tourists whose trips to the study site are less subjected to holidays and weather. Third, the mobile phone data were used to only monitor visitation to the protected area so that the study results could not capture whether these weather variables can even change the public’s travel destinations (e.g. from a protected area to a shopping mall). Fourth, this study does not examine impacts of visitor changes by the tropic weather on wildlife and ecological conditions in the protected area (e.g. visitors may walk more slowly under hotter conditions and not get as deep in the reserve), indicating a need for future studies.

## Conclusion

This study applied mobile phone data to analyze impacts of tropical weather (temperature, rainfall, and wind) and holidays on recreational ecosystem services in an urban protected area in Singapore. The mobile phone data were used to identify visitor numbers, their stay time in the protected area, their home regions, as well as their ethnic groups. The study results demonstrated that rising temperatures below 31.7 °C slightly increased visitor numbers (1.9% per degree of temperature increase), potentially indicating the public’s existing tolerance to high temperature in Singapore. Meanwhile, these rising temperatures decreased visitors’ stay time in the protected area (1.4% per degree of temperature increase). However, rain and wind had limited impacts on visitors. Compared to the weather variables, moreover, holidays had bigger impacts on visitors, particularly on the Chinese ethnic group and those visitors living far away from the protected area. These holiday impacts imply that there are several factors affecting the public use of protected areas, in addition to weather conditions. The study revealed several advantages of mobile phone data application to analyzing weather impacts on public use of urban protected areas.

## Materials and methods

### Data collection

Mobile phone data were used to identify daily visitors to Bukit Timah in Singapore from May to December 2017 (Table 1, Figs. 1 and 2). Bukit Timah includes a nature reserve, which is under “Category IV: Habitat/Species Management Area”, according to the categories of protected areas from the International Union for Conservation of Nature (IUCN)^10^. As of 2017, Singapore had a high mobile phone penetration rate (about 150% of the total population)^65^. We purchased mobile phone data from the Singtel Group Company, the biggest mobile service provider in Singapore. The data were anonymized so that the researchers could not identify individual mobile phone users. The market share of the company in 2017 was about 49% in Singapore^64^. The data showed two types of public use of the protected area: (1) daily visitors and (2) their stay time in the protected area. First, the data allowed identifying a total of daily mobile phone users who stayed within the administrative boundary of the protected area longer than 15 min. This boundary was based on the subzone system of the Master Plan 2014 in Singapore. Second, the data showed the total stay time of these daily visitors in the protected area in minutes. Based on this information, we were able to calculate the average stay time per visitor. In addition to these total daily visitors, the mobile phone data allowed identifying how many of these visitors were from different regions (e.g. counts of visitors from Central, West, North, North-east, and East regions, as well as their total stay time in the protected area) and ethnic groups (e.g. counts of visitors who registered themselves as Chinese, Eurasian, Indian, and Malay Singaporeans in their mobile service subscription). The mobile phone data were able to indicate visitor home regions by observing their staying locations during the night. This information was used to identify and exclude those mobile phone users living in the subzone of the protected area. Unfortunately, mobile phone data only showed total visitor counts under certain categories (e.g. total visitors from all regions, total visitors from the East region, or total Chinese visitors) rather than providing panel data of the visitors (e.g. identifying a home region and ethnicity of individual visitors). To protect the privacy of mobile phone users, the mobile service provider coded the data counts as − 1 in case that visitor counts were less than 39. However, only 16 cases of − 1 existed in the data of Eurasian and Malay visitors. The other visitor counts were always higher than 39 since Bukit Timah is a popular recreational site in Singapore. These cases of − 1 were treated as missing data and excluded from the analyses of Eurasian and Malay visitors.

**Table 1:** Descriptive statistics of the study data. The variables were measured from May to December 2017.

Variables	Min	Max	Mean	SD
**Visitor information**				
Daily visitors (no.)	1,756.0	5,154.0	3,502.0	624.8
Daily average stay time per visitor (minutes)	155.0	329.0	240.5	30.9
**Weather of the protected area**				
Daily max. temperature (°C)	25.6	35.5	31.6	1.5
Daily mean wind speed (km/h)	3.2	13.3	7.0	2.0
Daily total rainfall (mm)	0.0	88.8	7.3	13.8
**Institutional seasonality**				
Holidays (1: yes, 0: no)	0	1	0.3	0.5

**Figure 4:**
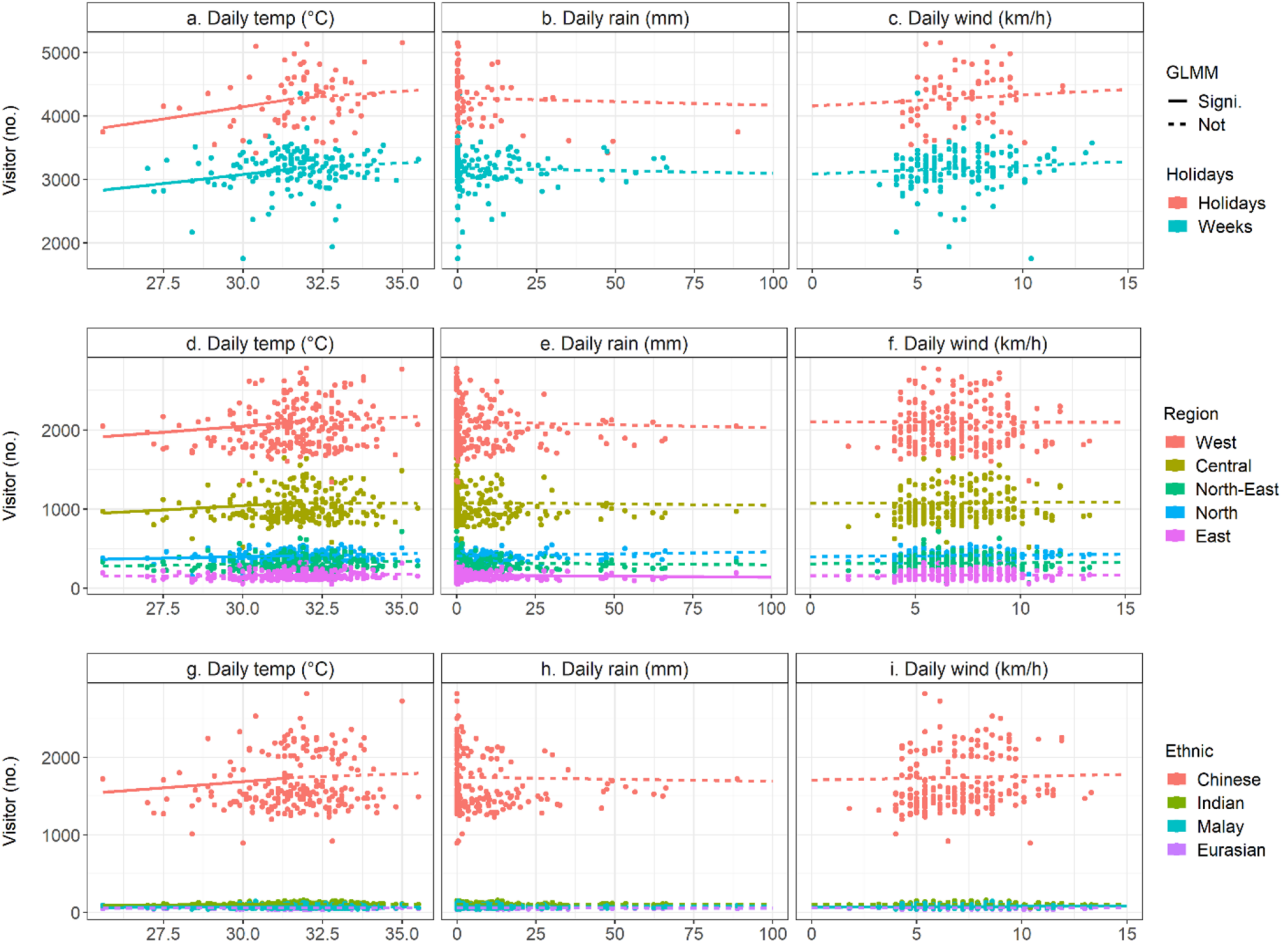
Distribution of visitor and weather variables. Visitors are grouped into all visitors (**a**–**c**), visitors by home regions (**d**–**f**), and visitors by their ethnic groups (**g**–**i**). The weather variables represent daily maximum temperature (°C), daily total rainfall (mm), and daily mean wind speed (km/h). GLMM shows regression lines from generalized linear mixed-effects models, statistically significant at lower than a 5% level (Signi.) and not significant (Not).

Weather data were collected from the National Environment Agency in Singapore (Fig. 4). These weather data included variables of daily maximum temperature (°C), daily total rainfall (mm), and daily mean wind speed (km/h) from May to December 2017. Although limited to represent all factors determining outdoor thermal comfort, these variables are still key factors strongly linked with outdoor thermal comfort^15^, outdoor activities in Singapore^18,19,44^, and Tourism Climatic Index^45,46^. The Tourism Climatic Index includes both daily maximum temperature (or a daytime comfort index) and daily mean temperature (or a daily comfort index). We only examined daily maximum temperature since Bukit Timah Nature Reserve is officially open to the public from 7 am to 7 pm. Further, research could examine hourly weather data using data loggers. These data were not available for our study location representing a limitation. Daily maximum and mean temperatures were also highly correlated. The weather data were collected from three weather stations close to Bukit Timah: Upper Peirce Reservoir, Clementi, and Newton stations. The Upper Peirce Reservoir station was primarily used for the analysis. Any missing data from the Upper Peirce Reservoir station were replaced with the data from either Clementi station (about 1.8 km from the study site) or Newton station (about 6.2 km from the study site).

**Figure 5:**
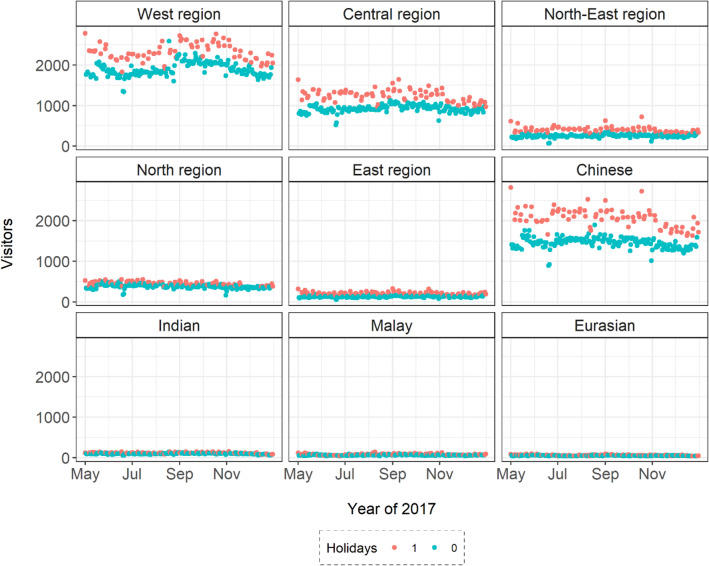
Daily visitors to Bukit Timah from May to December 2017. Visitors are grouped by their home regions and ethnic groups. The figure legend shows visitor numbers during holidays (1) and weekdays (0).

Holidays including weekends were included in this study as an institutional seasonality variable^50^. It is well known that holidays have significant impacts on tourism^50^ as well as nature-based outdoor activities^40^ so that this variable was used to represent a key social factor and to compare its impacts with the weather impacts. The variable was coded as a dummy variable: it had a value of 1 for public holidays and weekends from May to December 2017, while it had 0 for the rest of weekdays (Fig. 5).

### Statistical analyses

Generalized linear mixed-effects models were employed to test the influence of the weather variables on daily total visitors and their stay time in the urban protected area. Since the mobile phone data could only identify total visitors under certain categories (e.g. total visitors from all regions in Singapore, total visitors from East Region only, total Chinese visitors), a total of 10 models were established to test the weather impacts on these diverse sociodemographic factors (Fig. 5). The models were categorized into two groups following the research questions. Group 1 models were used to analyze weather impacts on total daily visitors to the protected area. Group 2 models were used to examine weather impacts on visitors’ stay time in the area. In all models, weather and holiday variables were tested as fixed effects, while month was used as a random intercept to account for temporal autocorrelation (Fig. 5). All models utilized a negative binomial distribution using a log link function. The models did not present multicollinearity as variance inflation factors of the variables were less than 1.2. The Spearman’s rank correlations among the variables were also less than 0.32 in absolute values^66^.

A piecewise regression approach^67^ was adopted into all the generalized linear mixed-effects models to examine the variable of daily maximum temperature since several studies indicate that different tolerance thresholds might exist with different temperature ranges^68,69^. To determine a breakpoint of the temperature variable for the piecewise regressions, we analyzed multiple models with a range of temperatures from 28 to 34 °C with an interval of 0.1 °C. 31.7 °C was selected as the breakpoint as it supported the lowest Akaike Information Criterion of the models. This breakpoint was also supported by a study that a maximum tolerable outdoor temperature in Singapore is rather high^18^. Confidence intervals of the piecewise variable were calculated by the delta method. All model estimations were based on the R package glmmTMB^70^. All statistical analyses of the study were conducted using R 3.4.1^53^.
